# Possible effect of norepinephrine transporter polymorphisms on methylphenidate-induced changes in neuropsychological function in attention-deficit hyperactivity disorder

**DOI:** 10.1186/1744-9081-8-22

**Published:** 2012-05-16

**Authors:** Subin Park, Jae-Won Kim, Young-Hui Yang, Soon-Beom Hong, Min-Hyeon Park, Boong-Nyun Kim, Min-Sup Shin, Hee-Jeong Yoo, Soo-Churl Cho

**Affiliations:** 1 Division of Child and Adolescent Psychiatry, Department of Psychiatry, Seoul National University College of Medicine, Seoul, Republic of Korea; 2 Department of Psychiatry, Pusan National University YangSan Children's Hospital, Pusan, Republic of Korea; 3 Department of Neuropsychiatry, Seoul National University Bundang Hospital, Seongnam, Republic of Korea; 4 Division of Child and Adolescent Psychiatry, Department of Psychiatry, Seoul National University College of Medicine, 101 Daehak-No, Chongno-Gu, Seoul, South Korea

**Keywords:** SLC6A2, Polymorphism, Continuous performance test, Methylphenidate

## Abstract

**Background:**

Dysregulation of noradrenergic system may play important roles in pathophysiology of attention-deficit/hyperactivity disorder (ADHD). We examined the relationship between polymorphisms in the norepinephrine transporter *SLC6A2* gene and attentional performance before and after medication in children with ADHD.

**Methods:**

Fifty-three medication-naïve children with ADHD were genotyped and evaluated using the continuous performance test (CPT). After 8-weeks of methylphenidate treatment, these children were evaluated by CPT again. We compared the baseline CPT measures and the post-treatment changes in the CPT measures based on the G1287A and the A-3081T polymorphisms of *SLC6A2*.

**Results:**

There was no significant difference in the baseline CPT measures associated with the G1287A or A-3081T polymorphisms. After medication, however, ADHD subjects with the G/G genotype at the G1287A polymorphism showed a greater decrease in the mean omission error scores (p = 0.006) than subjects with the G/A or A/A genotypes, and subjects with the T allele at the A-3081T polymorphism (T/T or A/T) showed a greater decrease in the mean commission error scores (p = 0.003) than those with the A/A genotypes.

**Conclusions:**

Our results provide evidence for the possible role of the G1287A and A-3081T genotypes of *SLC6A2* in methylphenidate-induced improvement in attentional performance and support the noradrenergic hypothesis for the pathophysiology of ADHD.

## Background

Attention-deficit hyperactivity disorder (ADHD) is a disorder primarily characterised by inattention, impulsivity, and hyperactivity, with a worldwide prevalence of 5.3% [1]. It has an estimated heritability of approximately 76 percent and is thought to be a complex, polygenic disorder [2].

Although the aetiology of ADHD is not fully understood, there is evidence that dysregulation of the central noradrenergic system and the dopaminergic system may be involved in the pathophysiology of ADHD [3,4]. The central noradrenergic system is involved in the modulation of attention, working memory, and response inhibition, and it has been suggested that norepinephrine improves attention by increasing the signal-to-noise ratio of dopamine-containing neurons, particularly in the prefrontal cortex [5-7]. The hypothesis that the noradrenergic system is involved in ADHD has been largely driven by the understanding that medications for the disorder have drug targets in the catecholamine neurotransmitter system. It has been suggested that methylphenidate (MPH), when administered for the treatment of ADHD, acts by inhibiting the norepinephrine transporter (NET) [8] and the dopamine transporter [9] and stimulating the noradrenergic alpha2 receptor and the dopamine D1 receptor [9]. The clinical efficacy of atomoxetine, a highly selective noradrenergic reuptake inhibitor, in treating ADHD patients is also consistent with noradrenergic involvement in the pathophysiology of ADHD [10,11].

The gene (*SLC6A2*) that codes for NET, located on chromosome 16q12.2, is a candidate gene for association with ADHD [12-14]. A-3081T single nucleotide polymorphism (SNP) (rs28386840) in the promoter region and a G1287A SNP (rs5569) in exon 9 are the two major polymorphisms investigated in relation to this disorder; however, the findings are inconsistent. Kim et al. [15] demonstrated that the T allele at the -3081 polymorphism was associated with ADHD and significantly decreased promoter function, as compared to the A allele. Joung et al. [16] also found an association between the presence of the T allele at the -3081 polymorphism and the occurrence of ADHD in a Korean population. According to a study by Yang et al. [17], the A/A genotype at the G1287A polymorphism was associated with a poor response to MPH treatment in a Chinese ADHD population. Although several other studies that followed did not find a significant association between the A-3081T or G1287A polymorphisms and ADHD [18-20], recent studies that examined Korean children with ADHD found associations between the G/G genotype at the G1287A polymorphism [21] or the presence of the T allele at the -3081 polymorphism [22] and an adequate response to MPH treatment.

Noradrenergic dysfunction related to norepinephrine transporter-mediated mechanisms may be associated with cognitive impairments in ADHD. The continuous performance test (CPT) is one of the most widely used neuropsychological tests in ADHD. The test assesses several aspects of attentional performance, including sustained attention in response to target stimuli and inhibitory control in response to non-target stimuli [23]. A meta-analytic review by Frazier et al. [24] reported that the CPT measures possess the largest effect size for the diagnosis of ADHD, and the measures of CPT have recently been proposed as a promising endophenotype for ADHD [25]. A number of studies, most of which focused on dopaminergic genes [26], were designed to investigate the genetic basis of CPT measures. In contrast, only a few studies have explored the association between NET genes and CPT measures. Kollins et al. [25] reported an association between a *SLC6A2* SNP (rs3785155) and response time variability of the CPT in 364 individuals from 152 families with at least one child diagnosed with ADHD. However, in a study by Cho et al. [18], there were no significant associations between genotypes of the G1287A and the A-3081T polymorphisms and the CPT measures. More recently, Song et al. [27] reported that subjects with the homozygous G/G genotype at the G1287A SNP showed significantly lower commission errors than subjects without the G/G genotype.

Considering the associations between NET genes and the response to MPH treatment or CPT measures previously reported, we propose that there may be an association between the polymorphisms of *SLC6A2* and MPH-induced changes in CPT. To our knowledge, there have been no studies of the association between *SLC6A2* and MPH-induced changes in CPT. An examination of such an association would inform the roles of the genetic polymorphisms of *SLC6A2* in the effectiveness of MPH treatment on core deficits of ADHD and further clarify the roles of the noradrenergic systems in the genetic basis of ADHD. Thus, we examined the relationship between the presence of the G1287A or A-3081T polymorphism within the *SLC6A2* gene and CPT measurements before and after 8 weeks of MPH treatment in children with ADHD.

## Methods

### Subjects

We recruited 53 children with ADHD from a child psychiatric clinic at Seoul National University Hospital in South Korea. Inclusion criteria were 1) diagnosis with ADHD according to the DSM-IV criteria, as ascertained by a child psychiatrist; and 2) age 6–18 years. Exclusion criteria were 1) any other mental disorders except for mild oppositional defiant disorder and anxiety disorder not requiring medication; 2) a past or present history of neurological illness; 3) an IQ below 70; 4) learning disabilities; 5) any history of substance abuse; and 6) previous treatment with MPH. All participants were drug-naïve at the time of study entry. To diagnose ADHD and any comorbid disorders, we used the Korean Kiddie-Schedule for Affective Disorders and Schizophrenia-Present and Lifetime Version (K-SADS-PL) [28]. We assessed intellectual abilities using the Korean version of the Wechsler Intelligence scale for Children (KEDI-WISC) [29].

### Procedures

Before medication, parents of participants completed an attention-deficit hyperactivity disorder rating scale (ADHD-RS), and participants underwent CPTs. Subjects received 0.35 ~ 1.77 mg/kg/day MPH (either extended-release MPH or osmotic release oral system MPH) for 8 weeks. Doses were adjusted depending on each patient's symptom severity and drug tolerability. The mean dose of MPH was 0.86 (SD 0.29) mg/kg/day. The maximum dose was 54 mg/day. All subjects took medications once per day in the morning. After 8 weeks’ MPH treatment, the ADHD subjects underwent the CPT again. On the day of the CPT, patients were instructed to take MPH in the morning as usual.

This study was conducted as part of a Health & Medical Technology R&D program and was approved by the institutional review board (IRB) for human subjects at the Seoul National University Hospital. Parents/guardians provided written informed consent, and the children provided verbal assent to participate in this study.

### Neuropsychological assessments

We used a computerised CPT [23] to measure the neuropsychological functions of the children with ADHD. The Korean version of the CPT has been standardised, and its validity and reliability are well established [30]. The four variables recorded were (1) omission errors (failure to respond to the target), which are commonly interpreted as a measure of inattention; (2) commission errors (responding inappropriately to the non-target), which are commonly interpreted as a measure of impulsivity; (3) response times for correct responses to the target, which are interpreted as a measure of information processing and motor response speed; and (4) the standard deviation of the response times for correct responses to the target (response time variability), which is interpreted as a measure of variability or consistency of attention.

### Genotyping

Genomic DNA was extracted from whole blood lymphocytes using a G-DEXTM II Genomic DNA Extraction Kit (Intron, Korea). The detection of a single nucleotide polymorphism was based on an analysis of primer extension products generated from previously amplified genomic DNA, using a chip-based matrix-assisted laser desorption/ionisation time-of-flight (MALDI-TOF) mass spectrometry platform (Sequenom, California, USA).

The *SLC6A2* polymorphisms were genotyped as previously described [15] with slight modifications. In brief, oligonucleotide primers [5’ - ACG TTG GAT GAG ACC CTA ATT CCT GCA CCC and 5’ - ACG TTG GAT GTT CAG GAC CTG GAA GTC ATC for the G1287A polymorphism] were used to generate polymerase chain reaction (PCR) products. The PCR was performed in a volume of 5 μl containing 1 X PCR buffer (TAKARA, Japan), 2.5 mM MgCl_2_, 0.2 mM of each dNTP, 0.1 U HotStarTaq Polymerase (Qiagen, Germany), 8 pM of each primer, and 4.0 ng of genomic DNA. The program consisted of denaturation at 95°C for 15 min; followed by 45 cycles at 95°C for 20 sec, 56°C for 30 sec, and 72°C for 1 min; with a final extension at 72°C for 3 min. Following the PCR, unincorporated dNTP was removed by the addition of 0.3 U of shrimp alkaline phosphatase and incubation for 20 min at 37°C, followed by 5 min at 85°C for enzyme inactivation. The total volume of each reaction was 9 μl, including hME enzyme (Thermo Sequenase, GE Healthcare, UK), ACT termination mix, and 5 μM of extension primer. The primer extension protocol was started at 94°C for 2 min, followed by 55 cycles at 94°C for 5 sec, 52°C for 5 sec, and 72°C for 5 sec. After desalting the reaction products with SpectroCLEAN (Sequenom), samples were analysed in the fully automated mode with a MALDI-TOF MassARRAY system (Bruker-Sequenom, California, USA).

### Statistical analysis

Allele frequency was determined, and the Hardy-Weinberg equilibrium was calculated using a goodness-of-fit *χ*^2^ test.

Due to the small number of individuals with the A/A genotype for the G1287A polymorphism, the subjects were dichotomized according to whether or not they possessed the rare A allele (recessive model). For the A-3081T polymorphism, which follows a co-dominant inheritance pattern, subjects were dichotomized according to whether or not they possessed the T allele, and this methodology was based on our previous study that found an improved response to MPH treatment in ADHD subjects with the T allele as compared to those without [22].

Group differences in the clinical variables involving continuous data were computed using an independent two sample *t*-test. Between-group comparisons involving categorical data were assessed using the *χ*^2^ test or Fisher’s exact test. The association between the evaluated genotypes and the neuropsychological measurements was investigated using a one-way analysis of variance (ANOVA) or independent two sample t-tests. Between-group differences according to genotype were assessed based on the percent change in the CPT measurements [$\frac{\left(post-treatment\;CPT\;score-baseline\;CPT\;score\right)}{baseline\;CPT\;score}*100$], rather than on inherent changes, to exclude the influence of baseline CPT scores on the MPH-induced changes to CPT scores. All statistical analyses were performed using SPSS (version 12.0; SPSS Inc., Chicago, IL). The significance level was set at p = 0.05/2(SNPs)*4(outcome measures) = 0.006.

## Results

Of the 53 subjects in this study, 45 (84.9%) were boys and 8 (15.1%) were girls. According to the DSM-IV criteria, the combined subtype is the most common (N = 36, 67.9%), followed by the inattentive (N = 14, 26.4%) and hyperactive-impulsive (N = 3, 5.7%) subtypes.

The G1287A genotype analysis of *SLC6A2* revealed that the G/G genotype was observed in 31 subjects (58.5%), the A/G genotype in 19 subjects (35.8%), and the A/A genotype in 3 subjects (5.7%). The A-3081T genotype analysis of *SLC6A2* revealed that the T/T genotype was observed in 12 subjects (18.9%), the A/T genotype in 28 subjects (52.8%), and the A/A genotype in 13 subjects (24.5%). The distributions of the genotypes for the G1287A and the A-3081T polymorphisms were in agreement with the expected values of the Hardy-Weinberg equilibrium (p > 0.99 and p = 0.790, respectively).

Table 1 shows the demographic and clinical characteristics of the ADHD subjects according to their G1287A and A-3081T genotypes. When dichotomised according to whether the subjects have the rare allele or not (G/G vs. A/G + A/A for G1287A and A/A vs. A/T + T/T for A-3081T), there were no significant group-differences in age, gender, intelligence, frequency of subtype, or score of ADHD-RS.

**Table 1:** **Demographic and clinical characteristics of ADHD subjects according to G1287A and A-3081T genotypes of*SLC6A2***

	**G1287A**				**A-3081T**			
	**GG (n = 31)**	**AG + AA (n = 22)**			**AA (n = 13)**	**AT + TT (n = 40)**		
	**N (%)**	**N (%)**	***X***^**2**^	**p-value**	**N (%)**	**N (%)**	***X***^**2**^	**p-value**
Sex			0.06	>0.99			0.39	0.662
Male	26 (83.9)	19 (86.4)			12 (92.3)	33 (82.5)		
Female	5 (16.1)	3 (13.6)			1 (7.7)	7 (17.5)		
ADHD subtype			4.67	0.097			3.87	0.145
Combined	23 (74.2)	13 (59.1)			6 (46.2)	30 (75.0)		
Inattentive	8 (25.8)	6 (27.3)			6 (46.2)	8 (20.0)		
Hyperactive-impulsive	0 (0)	3 (13.6)			1 (7.7)	2 (5.0)		
Comorbidity								
ODD	2 (6.5)	4 (18.2)	1.76	0.219	0	6 (15.0)	2.20	0.317
Anxiety disorder	4 (12.9)	3 (13.6)	0.01	>0.99	1 (7.7)	6 (15.0)	0.46	0.667
Tic disorder	1 (3.2)	1 (4.5)	0.06	>0.99	1 (7.7)	1 (2.5)	0.73	0.434
Enuresis	2 (6.5)	0	1.48	0.505	1 (7.7)	1 (2.5)	0.73	0.434
	Mean (SD)	Mean (SD)	t	p-value	Mean (SD)	Mean (SD)	t	p-value
Age	9.01 (1.96)	9.12 (1.99)	−0.19	0.848	9.26 (2.06)	8.45 (1.47)	1.31	0.198
IQ	108.74 (14.25)	109.27 (11.54)	−0.14	0.886	109.42 (14.90)	108.83 (12.69)	0.16	0.876
ADHD-RS, total	28.45 (10.60)	28.18 (9.97)	0.09	0.926	24.92 (13.28)	29.45 (8.98)	−1.40	0.169
Inattentive	16.13 (6.38)	15.27 (5.22)	0.52	0.607	14.31 (6.92)	16.25 (5.53)	−1.03	0.306
Hyperactive-impulsive	12.32 (5.49)	12.91 (6.52)	−0.35	0.725	10.62 (7.58)	13.20 (5.18)	−1.39	0.171
Mean dose, mg/kg	0.86 (0.33)	0.86 (0.25)	−0.01	0.996	0.78 (0.31)	0.89 (0.28)	−1.10	0.277

Abbreviations: *ADHD* attention-deficit hyperactivity disorder, *ADHD-RS* ADHD-Rating Scale.

**Figure 1:**
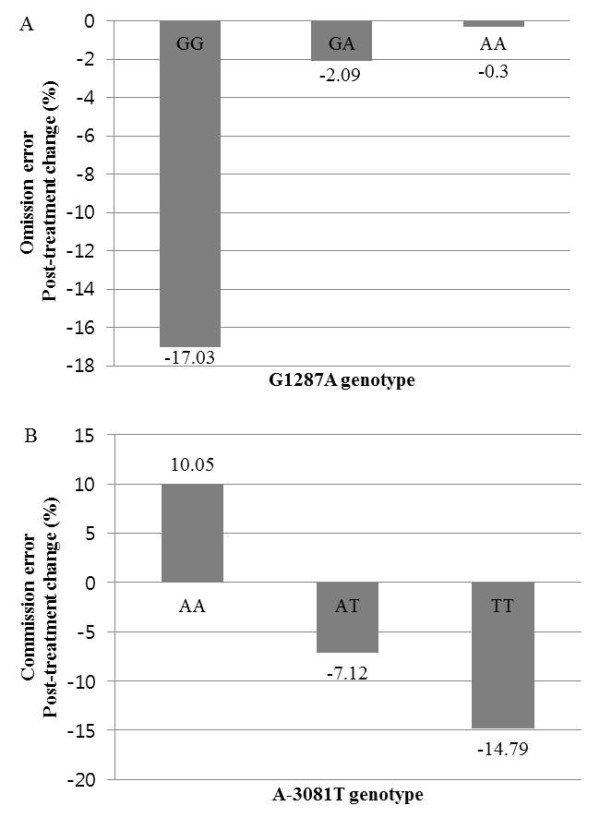
**Post-treatment change (%) in continuous performance test measurements according to the presence of the (A) G1287A genotypes and (B) A-3081T genotypes.** Only the significant results are displayed.

There were no significant group differences in the baseline scores on the CPT according to the G1287A and the A-3081T genotypes (Tables 2 and 3). However, after 8 weeks’ treatment with MPH, subjects with the G/G genotype at the *G1287*A polymorphism showed more improvement in the mean omission error scores (p = 0.006) than those with the A/G or A/A genotypes. Subjects with the G/G genotype showed the greatest decrease in omission errors, flowed by those with the A/G genotype and the A/A genotype (p = 0.023), although the small number of subjects with the A/A genotype limited statistical comparisons among the three genotypes (Table 2, Figure 1A). Subjects with the A/A genotype at the A-3081T polymorphism showed less improvement in the mean commission error scores (p = 0.003) than those with the A/T or T/T genotypes. Subjects with the T/T genotype showed the greatest decrease in commission errors, followed by the A/T genotype and A/A genotype (p = 0.007). Actually, the numbers of commission errors of subjects with the A/A genotype increased after treatment, while those of subjects with other genotypes decreased (Table 3, Figure 1B).

**Table 2:** **Baseline neuropsychological characteristics and post-treatment changes according to the G1287A genotype of*SLC6A2***

	**GG (=31)**	**GA(n = 19)**	**AA(n = 3)**		**GG (n = 31)**	**GA + AA (n = 22)**		
	**Mean (SE)**	**Mean (SE)**	**Mean (SE)**	**p-value**	**Mean (SE)**	**Mean (SE)**	**Difference (95% CI)**	**p-value**
Baseline								
Omission errors	66.87(4.18)	61.16(4.68)	52.00 (4.62)	0.421	66.87(4.18)	59.91 (4.12)	6.96 (-5.20-19.12)	0.256
Commission errors	54.97 (3.44)	61.74 (6.61)	53.00 (7.55)	0.567	54.97 (3.44)	60.55 (5.75)	−5.58 (-18.27-7.12)	0.382
Response time	58.52 (2.98)	54.95 (3.40)	55.67 (2.85)	0.728	58.52 (2.98)	55.05 (2.94)	3.47 (-5.20-12.14)	0.425
Response time variability	66.26 (2.77)	64.95 (4.85)	67.00 (8.02)	0.961	66.26 (2.77)	65.23 (4.27)	1.03 (-8.75-10.82)	0.833
Post-treatment change (%)^a^								
Omission errors	−17.03 (3.25)	−2.09 (4.96)	−0.30 (4.19)	0.023	−17.03 (3.25)	−1.85 (4.3)	−15.19 (-25.81 to -4.57)	0.006
Commission errors	−5.89 (3.74)	−3.23 (5.47)	−0.8 (1.83)	0.870	−5.89 (3.74)	−2.90 (4.72)	−2.98 (-14.95-8.99)	0.619
Response time	0.35 (6.16)	−13.19 (6.23)	−6.33 (7.20)	0.338	0.35 (6.16)	−12.26 (5.45)	12.61 (-4.75-29.96)	0.151
Response time variability	−6.89 (3.11)	−4.39 (2.96)	−6.70 (5.27)	0.678	−6.89 (3.11)	−4.70 (2.62)	−5.09 9-16.93-6.75)	0.392

^a^$\frac{\left(post-treatment\;CPT\;score-baseline\;CPT\;score\right)}{baseline\;CPT\;score}*100$.

**Table 3:** **Baseline neuropsychological characteristics and post-treatment changes according to the A-3081T genotype of*SLC6A2***

	**AA (n = 13)**	**AT (n = 28)**	**TT (n = 12)**		**AA (n = 13)**	**AT + TT (n = 40)**	**Difference (95% CI)**	
	**Mean (SD)**	**Mean (SD)**	**Mean (SD)**	**p-value**	**Mean (SD)**	**Mean (SD)**	**Difference (95% CI)**	**p-value**
Baseline								
Omission errors	64.69 (7.00)	61.75 (3.63)	68.42 (7.14)	0.677	64.69 (7.00)	63.75 (3.31)	0.94 (-13.16-15.05)	0.894
Commission errors	51.54 (3.79)	54.64 (3.39)	69.67 (10.02)	0.088	51.54 (3.79)	59.15 (3.91)	−7.61 (-22.10-6.88)	0.297
Response time	54.15 (4.19)	58.36 (2.47)	57.25 (6.08)	0.727	54.15 (4.19)	58.02 (2.47)	−3.87 (-13.81-6.06)	0.438
Response time variability	69.15 (7.74)	64.46 (2.18	75.42 (4.32)	0.727	69.15 (7.74)	64.75 (1.97)	4.40 (-6.74-15.55)	0.431
Post-treatment change (%)^a^								
Omission errors	−14.12 (5.7)	−8.75 (3.78)	−11.67 (6.27)	0.728	−14.12 (5.7)	−9.63 (3.21)	−4.49 (-17.54-8.55)	0.493
Commission errors	10.05 (8.04)	−7.12 (2.92)	−14.79 (4.52)	0.007	10.05 (8.04)	−9.42 (2.49)	19.48 (6.88-32.09)	0.003
Response time	−15.82 (6.81)	1.26 (6.00)	−7.38 (10.27)	0.259	−15.82 (6.81)	−1.33 (5.18)	−14.48 (-34.35-5.39)	0.150
Response time variability	−13.12 (5.39)	−2.45 (2.33)	−6.48 (4.42)	0.150	−13.12 (5.39)	−3.66 (2.09)	−11.52 (-24.79-1.74)	0.087

^a^$\frac{\left(post-treatment\;CPT\;score-baseline\;CPT\;score\right)}{baseline\;CPT\;score}*100$.

## Discussion

We found significant associations between MPH-induced changes in omission errors of the CPT and the NET G1287A genotype as well as between MPH-induced changes in commission errors of the CPT and the NET A-3081T genotype in Korean children with ADHD. Patients with the G/G genotype at the G1287A polymorphism showed a greater improvement in omission errors of the CPT than those with the G/A or AA genotype, and patients with the T allele as one of the alleles (A/T or T/T genotypes) at the A-3081T polymorphism showed a greater improvement in commission errors of the CPT than those with the A/A genotype.

No differences in pre-treatment CPT performance were associated with the G1287A or the A-3081T genotypes, consistent with our previous study [18]. However, our finding is inconsistent with the recent study by Song et al.[27], which reported that subjects with the G/G genotype at the G1287A polymorphism showed significantly lower commission errors than those without the G/G genotype (p = 0.026). The alpha level of their study (p < 0.05) was less conservative than that of our study using the Bonferroni correction (p < 0.006).

The omission errors are an indicator of deficits in sustained attention in response to target stimuli. Our data showed that the G1287A polymorphism may affect the outcome of MPH treatment on sustained attention deficits in ADHD, with the G/G genotype being associated with the greatest MPH-induced decrease in omission errors. This result with MPH is consistent with the results reported by Yang et al. [17], which indicated that Chinese ADHD subjects with the G allele (G/A or G/G genotypes) showed more symptom improvement in response to MPH than those with the A/A genotype, and the results of Song et al. [21], which showed that Korean ADHD subjects with the G/G genotype evinced more symptom improvement than those with the A allele (G/A or A/A genotypes). Together, these findings suggest a possible adventitious effect of the G allele in MPH-induced improvement of inattention, whether symptomatic or neuropsychological, in ADHD.

Despite the possibility of a role of the G1287A polymorphism in treatment response, a previous association study found no evidence of a biased transmission of any of the alleles of the G1287A polymorphism in a sample of Korean ADHD probands [18]. In addition, G1287A is a silent mutation that does not encode protein variants [31], and we cannot assume that G1287A is linked to alleles that have an effect on NET expression. However, G1287A is located within 5 kb from SNPs such as rs3285157, rs998424, and rs11568324, which are known to be associated with ADHD and in which linkage disequilibrium is very high (D0 = 0.96–1.0) [32]. Thus, it is possible that the association between G1287A polymorphism and the MPH-induced changes in omission errors results from the high linkage disequilibrium with these SNPs.

The commission errors are an indicator of deficits in response inhibition, which is considered to be the core deficit in ADHD [33,34]. Our data showed that the A-3081T polymorphism may affect the results of MPH treatment on response inhibition in ADHD, with the T allele being associated with an MPH-induced decrease in commission errors. In terms of response to MPH treatment, this result is consistent with a previous Korean study in which ADHD subjects with the T allele (A/T or T/T genotype) showed more symptom improvement in response to MPH than those with the A/A genotype [22]. Unexpectedly, the numbers of commission errors for subjects with the A/A genotype increased following MPH treatment. However, these increases were not statistically significant (p = 0.360), and this result should be interpreted with caution due to the small sample size.

In previous studies, the frequency of the T allele at the A-3081T polymorphism was significantly higher in ADHD subjects than in controls [15,16], and this allele significantly decreased promoter function compared with the A allele [15]. Downregulated promoter function of *SLC6A2* and the consequent decrease in transcriptional activity in ADHD subjects with the T allele at the -3081 polymorphism, as reported by Kim et al. [15], may result in low levels of NET [33,34]. Our finding that MPH induced more improvement in inhibitory control deficits in subjects with the T allele at the -3081 polymorphism than in those without it may be explained by reduced levels of NET within the brains of subjects with the T allele at the -3081 polymorphism because the NET-blocking effect of MPH may be more prominent when the levels of NET are low. However, catecholamine-degrading enzymes like catecholamine-O-methyltransferase (COMT) or monoamine oxidase (MAO) may degrade any catecholamine that is produced by the NET-blocking effects of MPH. Therefore, the interaction effects of the NET and COMT/MAO polymorphisms on the response to MPH treatment should be investigated in further research. A specific improvement in inhibitory control by MPH may be explained by the therapeutic actions of MPH being associated with the preferential activation of noradrenergic and/or dopaminergic neurotransmission within the prefrontal cortex, which is the brain area known to mediate response inhibition [35-40]. Further studies using imaging and genetic approaches will be required to verify the hypothesis that the preferential action of MPH in the prefrontal region is associated with differences in improvement in inhibitory control deficits related to the A-3081T genotypes.

Several limitations may have influenced the findings in this study. First, the sample size of the present study is relatively small for genotypic analysis, so the results cannot be applied to the general population and should be interpreted carefully. Second, although the levels of performance of CPT and the frequencies of the NET polymorphisms in this study are similar to those in previous reports, we did not compare the CPT performances and NET polymorphisms of ADHD subjects and controls. Third, our study assessed CPT only after short-term MPH treatment outcomes. The MPH-induced neuropsychological changes produced by 8 weeks of MPH therapy may not be the same as long-term MPH-related changes. Forth, MPH was administered with no control of adherence by investigators. Finally, only three of the patients had the minor allele of the G1287A polymorphism, and this prevented precise statistical results.

## Conclusions

This preliminary study provides evidence for the possible roles of the G1287A and A-3081T genotypes of SLC6A2 in MPH-induced improvement in attentional performance and supports the noradrenergic hypothesis of the pathophysiology of ADHD. Further studies using larger sample sizes, controls, and long-term MPH treatment in the study designs should help to elucidate treatment-related neuropsychological changes related to genetic polymorphisms.

## Abbreviations

ADHD: Attention-deficit hyperactivity disorder; MPH: Methylphenidate; NET: Norpeinephrine transporter; SNP: Single nucleotide polymorphism; CPT: Continuous performance test; ADHD-RS: Attention-deficit hyperactivity disorder rating scale; COMT: Catecholamine-degrading enzymes like catecholamine-O-methyltransferase; MAO: Monoamine oxidase.

## Competing interests

None of the authors have any financial interest in the study, or any other conflict of interest.

## Authors’ contributions

SCC designed the study. SCC, JWK, BNK, and YHY participated in data collection. SBH and SP analyzed the data. SP prepared the first draft of the report. SCC and MSS supervised the statistical analysis. SP, SBH, MHP, and HJY interpreted the results. SP wrote the final report with input from all the authors. All authors read and approved the final manuscript.
